# Current Progress of Mitochondrial Genome Editing by CRISPR

**DOI:** 10.3389/fphys.2022.883459

**Published:** 2022-05-02

**Authors:** Tao Yin, Junjie Luo, Danqiong Huang, Hui Li

**Affiliations:** ^1^ Guangdong Engineering Research Center for Marine Algal Biotechnology, Guangdong Provincial Key Laboratory for Plant Epigenetics, Shenzhen Engineering Laboratory for Marine Algal Biotechnology, Longhua Innovation Institute for Biotechnology, College of Life Sciences and Oceanography, Shenzhen University, Shenzhen, China; ^2^ Key Laboratory of Optoelectronic Devices and Systems of Ministry of Education and Guangdong Province, College of Physics and Optoelectronic Engineering, Shenzhen University, Shenzhen, China; ^3^ Key Laboratory of Precision Nutrition and Food Quality, Department of Nutrition and Health, China Agricultural University, Beijing, China

**Keywords:** mitochondria, mtDNA, RNA import, CRISPR/Cas, gRNA

## Introduction

Human mitochondrial diseases are commonly caused by mutations in mitochondrial DNA (mtDNA). The severity of mitochondrial disease is associated with heteroplasmy, which is defined as the coexistence of two or more different mtDNA variants within one cell (Taylor and Turnbull, 2005). Although mitochondria-targeted zinc-finger nucleases (mitoZFNs) or mitochondria-targeted transcription activator-like effector nucleases (mitoTALENs) can be used for mitochondrial genome editing, they have limitations that include the laborious design and assembly of the monomers, their limited sequence specificity and large size. The CRISPR/Cas genome editing system is a powerful tool to precisely edit the genomes of a wide range of mammals and plants. However, the biggest challenge in utilizing this system in mitochondria is the delivery of exogenous guide RNA (gRNA) into mitochondria. Previous attempts at delivering gRNA via stem-loop motifs have been reported but there is no robust evidence to show the success of this approach. In the future, the efficient delivery of gRNA, with a mitochondrial localization signal (MLS), and the effective cleavage activity of the modified gRNA/Cas complex will be necessary for mitochondrial genome editing by CRISPR/Cas system.

## Current Methods for Mitochondrial Genome Editing

### Mitochondrial Genome and mtDNA Disorder

Mitochondria are double membrane-bounded organelles that are known as the “powerhouse of the cell.” They contain maternally inherited, double-stranded, multi-copy DNA of 16.5 kb. The genome is circular and encodes 13 protein subunits involved in the respiratory chain, 22 transfer RNAs (tRNAs) and two ribosomal RNAs (rRNAs) (Falkenberg et al., 2007).

Several mtDNA mutations have been associated with mitochondrial dysfunction (Wallace and Chalkia, 2013). As there are multiple copies of mtDNA within a cell, pathogenic mutations in human mtDNA are commonly heteroplasmic. When the percentage of mutated mtDNA molecules exceeds the threshold that compromises the mitochondrial function, thereby disrupting the overall cellular function, mtDNA disorder will occur (Vafai and Mootha, 2012). It has been reported that the A3243G mutation, at 50%–90% mtDNA heteroplasmy, may cause the severe mitochondrial encephalomyopathy, lactic acidosis and stroke-like episodes (MELAS) syndrome (Goto et al., 1990; Picard et al., 2014).

### Mitochondria-Targeted DNA Nucleases

**FIGURE 1:**
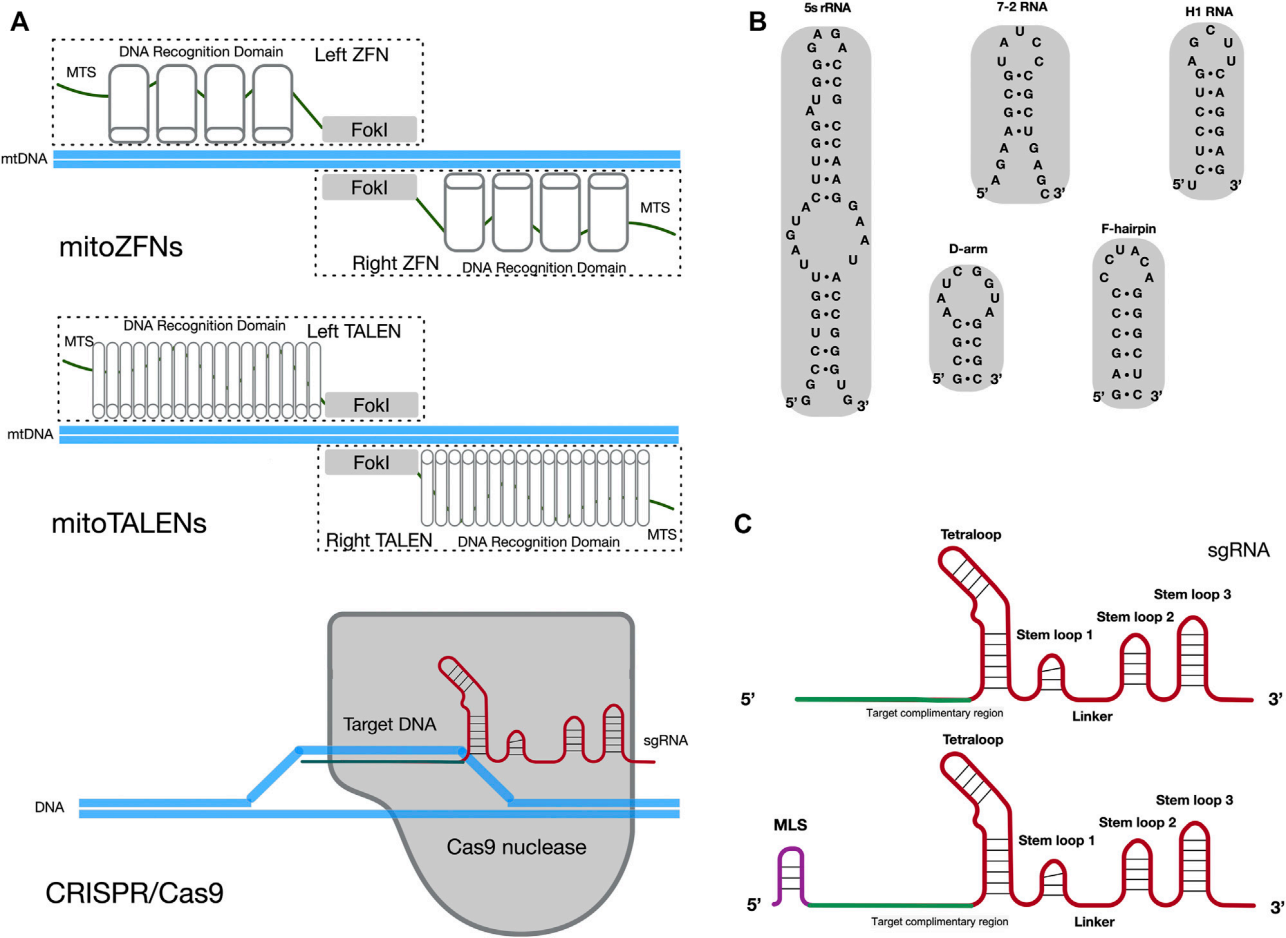
Potential methods for sgRNA importation into mitochondria. **(A)** Diagram illustrating the cleavage mechanism of mitoZFNs, mitoTALENs and the CRISPR/Cas9 system. MTS, mitochondrial targeting sequence; FokI, non-specific FokI nuclease; sgRNA, single guide RNA. **(B)** Stem-loop motifs identified in human 5s rRNA, 7-2 RNA, H1 RNA,D-arm and F-hairpin motifs identified in yeast tRNA^Lys (CUU)^. **(C)** The secondary structures of the original sgRNA and sgRNA with MLS. The original sgRNA is comprised of three stem loops and one tetraloop. When compared with the original sgRNA, the potential mitochondria-targeted sgRNA may be have an additional MLS at the 5′-end. The target complimentary region, which the RNA sequence for recognized target DNA, is shown in green. The backbone of the sgRNA is shown in red. MLS is the mitochondrial localization signal and is shown in purple.

For clinical applications that target mtDNA disorder, it is essential to achieve homoplasmic status in a cell by eliminating the mutated mtDNA. In contrast to nuclear DNA (nDNA) repair pathways, the efficient DNA double-strand break (DSB) repair and homologous recombination (HR) mechanisms are lacking in mammalian mitochondria (Nissanka et al., 2019; Carvalho et al., 2021). Once cut on both strands, mtDNA molecules are not repaired, which results in rapid degradation of mtDNA in mammalian cells (Peeva et al., 2018). Hence, several approaches have been developed to use mitochondria-targeted and site-specific DNA nucleases that will quickly degrade the mutant mtDNA in heteroplasmic cells, to adjust the heteroplasmy ratio (Patananan et al., 2016; Silva-Pinheiro and Minczuk, 2022). The expression of mitochondria-targeted restriction endonucleases (mtREs) has been used to shift the mtDNA heteroplasmy ratio from mutant to wild-type in the mouse model and patient somatic cells. Results indicated that mtREs specifically reduce the amount of mutated mtDNA molecules (Srivastava and Moraes, 2001; Tanaka et al., 2002). However, this mtRE approach has obvious limitations. For example, only one unique restriction site (XmaI) has been found to arise in approximately 200 different mtDNA mutations (Reddy et al., 2015). To overcome the limitation of mtREs, other alternative approaches have been developed. The use of mitoZFNs or mitoTALENs (Figure 1A), which are composed of a mitochondrial targeting sequence (MTS), the specific DNA recognition modules and a non-specific FokI nuclease, has successfully altered the heteroplasmy ratio in previous studies (Minczuk et al., 2008; Bacman et al., 2013; Gammage et al., 2014; Reddy et al., 2015; Gammage et al., 2016; Bacman et al., 2018; Gammage et al., 2018b). Zinc-finger proteins, as the recognition modules of the mitoZFN monomer, can recognize a 12 bp sequence. Unlike mitoZFN, the mitoTALEN monomer utilizes TAL effector proteins as the recognition modules that can recognize approximately 17 nucleotides. In addition to the conventional mitoTALEN monomer, a new monomer, mitoTev-TALE, has been successfully used to manipulate mutant mtDNA in cybrid cells (Pereira et al., 2018). The TAL effector proteins are linked to the I-TevI nuclease in mitoTev-TALE, rather than the FokI nuclease, as in mitoTALEN. Recently, a radically different approach for mtDNA editing has been reported, namely double-stranded DNA deaminase (DddA)-derived cytosine base editors (DdCBEs) (Mok et al., 2020; Lee et al., 2021; Silva-Pinheiro et al., 2022). The DdCBE is composed of mitoTALE proteins, the interbacterial toxin DddA and an uracil glycosylase inhibitor. It is able to precisely catalyze C•G-to-T•A conversions in human mtDNA with high target specificity.

Despite the promising results of mitoZFNs and mitoTALENs in mtDNA editing, these methods have limitations. Since each mitoZFN or mitoTALEN monomer needs to be designed and engineered to recognize a specific range of mtDNA sequences, they require enormous labor and cost-intensive protein production. In addition, the size of the coding nuclease sequences exceeds the capacity of current virus-based (adeno-associated virus, AAV) delivery systems (Moraes, 2014). The CRISPR/Cas9 genome editing system may solve this issue as it relies on the single guide RNA (sgRNA) to recognize a specific 20 bp DNA sequence and only Cas9 nuclease can cleave this specific DNA sequence (Figure 1A) (Hsu et al., 2013; Ran et al., 2013; Doudna and Charpentier, 2014). Currently, reliable chloroplast and mitochondrial genome editing methods by CRISPR/Cas9 system exist only in yeast and green algae (Yoo et al., 2020). Yoo et al. introduced plasmids, which contained a cassette for the organelle-specific expression of Cas9 nuclease and a cassette for the expression of sgRNA, into yeast mitochondria and *Chlamydomonas* chloroplasts through microprojectile transformation. The authors confirmed the insertion of donor DNA at the target sites of organelle DNA, facilitated by HR, only in the presence of Cas9/sgRNA activity. In mammals, two independent studies have attempted to edit mtDNA through the utilization of a CRISPR/Cas9 genome editing system (Jo et al., 2015; Bian et al., 2019). In these studies, mitochondria-targeted Cas9 nucleases (mitoCas9) and unmodified sgRNAs in cells resulted in mtDNA cleavage. These data implied that the original sgRNA, without any modifications, was spontaneously delivered into mitochondria from the cytosol. This occurred despite the double-membrane structure of mitochondria making it difficult for exogenous RNA and proteins to spontaneously enter mammalian mitochondria. However, attempts to repeat this work, by other research groups, have not been successful (Pereira and Moraes, 2017). Therefore, it is not clear whether the CRISPR/Cas9 genome editing system is able to cleave or edit mammalian mtDNA, due to the challenge of importing the exogenous sgRNA into the mitochondria.

In contrast to mitochondrial protein, the mechanism of importing nucleus-encoded RNA into mitochondria is still not fully understood (Gammage et al., 2018a). A controversial mechanism of importing RNA into mammalian mitochondria, mediated by polynucleotide phosphorylase (PNPase), is not widely accepted (Wang et al., 2010; Wang et al., 2012a). If the delivery of exogenous sgRNA into mitochondria can be achieved, the CRISPR/Cas9 genome editing system could be widely used to edit the mitochondrial genome.

### Delivery of Exogenous sgRNA Into Mitochondria

There are numerous endogenous RNAs reported to have functional roles in mammalian mitochondria, such as H1 RNA (RNase P), 7-2 RNA (RNase MRP) and 5S rRNA (Puranam and Attardi, 2001; Mercer et al., 2011; Smirnov et al., 2011). Notably, some distinct stem-loop motifs have been found at the 5′ or 3′ -end of these RNA molecules and could mediate the import of nucleus-encoded RNA molecules into mitochondria (Figure 1B). For example, it has been suggested that stem-loop motifs identified in H1 RNA and 5S rRNA are MLS for mitochondrial import of RNA molecules (Wang et al., 2012b; Towheed et al., 2014; Zelenka and Ježek, 2016). Meanwhile, other studies have claimed that the delivery of synthetic RNA into mammalian mitochondria can be achieved through the utilization of F-arm or D-hairpin motifs from yeast cytosolic tRNA^Lys (CUU)^ (Gowher et al., 2013; Tonin et al., 2014). In the CRISPR/Cas9 system, the sgRNA is created by fusing the CRISPR RNA (crRNA) and trans-activating crRNA (tracrRNA) sequences together into a single RNA chimera. It forms a T-shaped architecture, with one tetraloop and three stem-loops. Stem-loop 1 is essential for the formation of a functional Cas9/sgRNA complex. Stem-loop 2 and stem-loop 3 support the stable complex formation and enhance the stability of the sgRNA (Jinek et al., 2012; Anders et al., 2014; Nishimasu et al., 2014). Another study has suggested that sgRNA, with an elongated 5’ end or insertions in the tetraloop, should not significantly disrupt the CRISPR/Cas9 system (Tang et al., 2017).

The above studies provide some inspiration for methods of delivery of exogenous sgRNA into mitochondria. The potential mitochondria-targeted sgRNA architecture may be comprised of additional MLS at the 5′-end of the sgRNA and conserved structures in the sgRNA (three stem-loops and one tetraloop), to ensure that it is imported into mitochondria and to meet the requirement for Cas9/sgRNA complex formation, respectively (Figure 1C). The varied stem-loop motifs (20–40 nt RNA sequences) identified in mitochondria-targeted RNAs act as the MLS for sgRNA and may provide a potential pathway for importing sgRNA into mammalian mitochondria.

### Mitochondrial Genome Editing by CRISPR/Cas9 System

Attempts to develop a mitochondria-adapted CRISPR/Cas9 system, using mitoCas9 and sgRNA that contains the stem-loop motif, have been reported. One such study declared that the stem-loop motif from H1 RNA can mediate delivery of sgRNA into mitochondria. In mouse cells, modified sgRNA/mitoCas9 complexes were able to reduce the amount of mtDNA that carried the 11205G mutation in the mitochondrial ND4 gene (mtND4). Both modified sgRNAs, with the stem-loop motif from H1 RNA, and non-modified sgRNAs were present in the mitochondrial fraction. Modified sgRNAs were highly enriched in the mitochondria of transfected cells versus non-modified controls (Hussain et al., 2021). However, the process used in this study for mitochondria isolation did not remove the outer mitochondrial membrane to eliminate outer-membrane contaminants. Consequently, the levels of sgRNAs detected in the mitochondrial fraction may be over-estimated, due to contamination by cytosolic RNAs and RNAs associated with the outer membrane. In addition, analysis and quantification of mtDNA content, 48 and 72 h after cells were transfected, in the control and experimental groups, have not been performed in this study. The additional long-term analysis of mtDNA content may strengthen the conclusion that the mitochondria-adapted CRISPR/Cas9 system reduces the mitochondrial genomes.

Another study claimed that using a pair of modified sgRNAs, with an F-arm motif from yeast cytosolic tRNA^Lys (CUU)^, reduced the mtDNA content in Kearns Sayre Syndrome cybrids, through incorporation of mitoCas9. No changes in mtDNA content were observed when using a single modified sgRNA. Moreover, any significant shift of mtDNA heteroplasmy was not observed (Loutre et al., 2018). Loutre et al. detected either the modified sgRNAs, with an F-arm/D-hairpin, or original sgRNAs that lacked the stem-loop motif in the enriched mitochondrial RNA samples. The sgRNAs bearing the F-arm motif and the original sgRNAs had a similar ability (approximately 35%) to enter mitochondria. These data implied that mitochondria-adapted CRISPR/Cas9 system was unbale to specifically reduce mutant mtDNA content, and the F-arm motif was unable to improve the mitochondrial import of sgRNA. In addition, the cleavage activity of the sgRNA (with an F-arm or D-hairpin motif)/Cas9 complex had been partially or completely blocked, which meant that the stem-loop motif may have influenced the binding of sgRNA and Cas9 nuclease or the recognition process of the sgRNA and targeted DNA.

A different study found that numerous insertion/deletion (InDel) events in mtDNA were generated using the unmodified sgRNA/mitoCas9 complex (Wang et al., 2021). Interestingly, in this study, the InDel frequencies generated by the mitochondria-adapted CRISPR/Cas9 system were low, at <0.05%, which was much lower than the elimination efficacies of mutated mtDNA by mitoTALENs/ZFNs. Moreover, modified sgRNAs, with the stem-loop motif from 7-2 RNA or H1 RNA, were unable to increase the frequencies of InDel, when compared with the original sgRNA. These data implied that stem-loop motifs from 7-2 RNA and H1 RNA were unable to improve the import of sgRNA into mitochondria. Generally, homology between nuclear mitochondrial DNA sequences (NUMTs) and mtDNA causes problems for the detection of mtDNA variants from Next Generation Sequencing (NGS) data because the origin of sequences cannot be determined (Maude et al., 2019). Hence, the InDel events determined by NGS may be over-estimated in this study. Wang et al. need to prove that the observed InDel events happened in mtDNA and not in NUMTs. If this is not properly addressed, it will undermine the credibility of reports of CRISPR-mediated mtDNA manipulation.

### Mitochondrial Genome Editing by CRISPR/Cas12a System

In contrast to the CRISPR/Cas9 system, the CRISPR/Cas12a system only requires a single crRNA (42–44 nt) as gRNA (Zetsche et al., 2015; Fonfara et al., 2016). Recently, the use of a mitochondria-adapted CRISPR/Cas12a (mitoCRISPR/Cas12a) system for mtDNA manipulation has been reported (Antón et al., 2020). Antón et al. demonstrated that LbCas12a nucleases were localized to mitochondria with high efficiency and caused less mitochondrial damage than the other Cas nucleases, such as SpCas9 and SaCas9. These data suggested that LbCas12a had a much higher predisposition to be imported into mitochondria. In this study, unmodified sgRNA, sgRNA with the stem-loop motif from H1 RNA and sgRNA with the stem-loop motif from yeast tRNA^Lys (CUU)^ were detected in the nuclear fraction and, to a lesser extent, the mitochondrial fraction. These data showed that sgRNA can be imported into mitochondria regardless of whether an MLS was present. As with the Hussain et al. work, the levels of sgRNAs observed in mitochondria may be over-estimated, because the outer-membrane contaminants had not yet been fully removed from the mitochondrial fraction. Although the authors had not performed any assessment of the delivery efficiency of crRNAs (with or without an MLS) into mitochondria, they still tested the activity of mitoLbCas12a/crRNA (with or without the stem-loop motif from H1 RNA) complexes in the MELAS cybrids. In theory, mitoLbCas12a/crRNA complexes would reduce the mtDNA content. However, instead both modified and unmodified crRNAs seem to have increased the amount of mtDNA in a mitoLbCas12a-dependent manner. The authors did not explain the reasons for the mitoCRISPR/Cas12a system effects. These confusing results mean that it remains unclear whether the CRISPR/Cas12a genome editing system is able to edit mammalian mtDNA.

## Discussion

Although these reported data are not perfect and are unable to provide reasonable evidence to show that stem-loop motifs (D-hairpin, F-arm, 7-2 RNA and H1 RNA) enhance the delivery efficiency of gRNA (sgRNA and crRNA) into mammalian mitochondria, these attempts can provide many new perspectives on the import of gRNA. Meanwhile, several research reports have shown that nuclear non-coding RNAs (ncRNAs) act as important mediators of the crosstalk between the nucleus and the mitochondria. These ncRNAs could directly impact mitochondria by affecting transcripts of the mitochondrial genome (Kim et al., 2017; Vendramin et al., 2017). Further modification of the gRNA scaffold, by integrating the recent knowledge of mitochondrial ncRNA transport, may show promise for increasing the delivery efficiency of gRNA into mitochondria. The CRISPR screening strategy may efficiently identify appropriate architectures of gRNA with an MLS, for mitochondria-adapted CRISPR/Cas genome editing. For specific mutations associated with mtDNA disorder, the cleavage activity of the modified gRNA/Cas complex may require validation by exploration and confirmation *in vivo* and *in vitro*, as MLS may affect the binding of gRNA and Cas nucleases or the recognition of gRNA and targeted DNA sequences. Therefore, the utilization of potential MLS from mitochondria-targeted RNAs, for the import of gRNAs into mitochondria, can provide a potential future for mitochondrial genome editing by CRISPR/Cas system, which remains a relatively optimum strategy.

Human mtDNA mutations often affect mitochondrial function and cause mitochondrial diseases. Manipulation of the mutated mitochondrial genome is the most direct and thorough approach to resolve mitochondrial dysfunction. Currently, owing to the lack of an efficient delivery system to import gRNA into the mitochondria, the popular CRISPR/Cas genome editing method is still restricted to nuclear genomes and cannot efficiently be used to edit mitochondrial genomes. Early attempts to deliver gRNA into mitochondria were unsuccessful but may bring inspiration to follow-up researchers. Success at delivering exogenous gRNA into mammalian mitochondria may create new possibilities for mtDNA editing and will also help to prevent mitochondria disorder.
